# Examining the Director of Nursing Role in Long-Term Care: An Integrative Review

**DOI:** 10.1155/2023/8200746

**Published:** 2023-11-06

**Authors:** Julia Fineczko, Charlene H. Chu, Lisa Cranley, Aria Wills, Katherine S. McGilton

**Affiliations:** ^1^Lawrence S. Bloomberg Faculty of Nursing, University of Toronto, Toronto, ON, Canada; ^2^KITE Research Institute, Toronto Rehabilitation Institute-University Health Network, Toronto, ON, Canada; ^3^Rehabilitation Sciences Institute, University of Toronto, Toronto, ON, Canada; ^4^Institute for Life Course and Aging, University of Toronto, Toronto, ON, Canada

## Abstract

**Aim:**

To identify and examine the structures and processes that support the director of nursing role in long-term care homes.

**Background:**

The director of nursing in long-term care homes is central to overseeing and supporting the workforce and delivery of safe, quality resident care. With ongoing health human resource challenges and an aging population requiring care from long-term care homes, it is important to understand what individual and organizational factors support the director of nursing leadership in these settings. *Evaluation*. This review was guided by Cooper's five stages of the integrative review process. Donabedian's structure-process-outcome framework was applied to synthesize the literature. *Key Issue(s)*. Five individual-level structures (years of experience, level of education, demonstrated leadership capabilities, completed certification and/or established linkages with a professional association, and completed continuing education); three organizational-level structures (physical presence of leadership across the organization, a clear job description, and salary); and four processes (nursing home administrators and the director of nursing relationship, availability of onsite continuing education opportunities targeting directors of nursing and support for continuing education, cultivating relationships and enhancing networks beyond the long-term care home, and orientation to the role) were identified across 11 articles to support the director of nursing role in long-term care homes. *Conclusion(s)*. The findings indicate that there are individual characteristics that support the director of nursing in their role. Notably, there are organizational structures and processes that can be modified at the practice and policy level to better recruit, retain, and support the performance of directors of nursing. *Implications for Nursing Management*. There are actionable steps that leaders and decision-makers can take to support nursing leadership across long-term care homes and directly address health human resource challenges.

## 1. Introduction

### 1.1. Background

#### 1.1.1. The Long-Term Care Setting

Long-term care homes provide 24-hour health and personal care services to individuals with complex needs who do not require hospital level care but cannot be safely cared for in their own home [1]. Despite strict regulations within long-term care homes in North America, quality of care across homes remains an ongoing concern [2, 3]. The COVID-19 pandemic was an example of the shortcomings in long-term care homes globally creating an environment that is increasingly unpredictable, underresourced, and complex to lead [2, 4–8]. A public inquiry into the operations of the long-term care sector in Ontario, Canada, instigated by poor resident and family outcomes in the first wave of COVID-19, revealed a neglected system and called for immediate government attention and investment [2, 9, 10]; similar findings were also noted across long-term care homes in the United States [11] and Australia [12]. In European countries, factors contributing to the virus' spread in long-term care homes were inadequate staff training, lack of personal protective equipment, poor surveillance testing for both staff and residents who are at increased risk by virtue of their underlying health conditions, and the congregate type setting [13]. Critical to the success of implementing many of the recommendations outlined in these reports is the director of nursing, who alongside the nursing home administrator and medical director, is a member of the senior interprofessional leadership team with direct responsibility for the nursing department [14].

#### 1.1.2. The Director of Nursing Role

The director of nursing in long-term care homes is defined as a senior nurse leader who is responsible for planning, organizing, and directing nursing care in the nursing department. They are also a clinician expert, educator, collaborator, and advocate in the home and participate in quality improvement, emergency planning, risk management, regulation compliance, and advancing the health and safety of staff [3, 15].

Research demonstrates a link between the director of nursing role performance and staff satisfaction, retention, quality improvement, and overall performance in the home [16, 17]. Despite the reach and demonstrated importance of the role in the literature, there is a sustained history of long-term care homes experiencing vacancies or high turnover rates for this critical leadership position published in the literature that ranges from 24% to 147% a year [17, 18]. Factors influencing the director of nursing turnover include a disconnect between preferred and expected responsibilities [19], low satisfaction and low perception of autonomy/participation in decision-making, and working in a for-profit home [20]. Lower levels of tenure were also noted amongst directors of nursing with higher levels of education and those working in for-profit chain homes, as compared to directors of nursing working in not-for-profit homes or in for-profit nonchain homes [21]. The consequences of high turnover rates of the director of nursing role result in a disruption to the team and negative impact on the quality of care to residents [17, 22–24].

The director of nursing role may not be mandated in long-term care homes internationally; rather, the specific regulations and requirements for this role can vary depending on the governing bodies in different countries and regions [8, 25]. However, in Ontario, Canada, and across the US, the director of nursing role is mandated within the long-term care homes with minimum qualifications (i.e., being a Registered Nurse (RN) with at least one year of experience working in the sector, three years of experience working in a managerial/supervisory capacity in a healthcare setting, and demonstrated leadership and communication skills) with no expectation for continued education or formal leadership training [11, 14]. Two recent scoping reviews examined the characteristics of the nursing home administrator/director of nursing senior leadership role in long-term care homes and impact on quality outcomes [26, 27]. Both reviews found that the influence of the nursing home administrator/director of nursing role on quality is inadequately described in the literature, role preparedness in aged care is poorly defined and focused primarily on the nursing home administrator role, and quality outcomes mostly relate to care and to a lesser extent as an outcome to the leader and organization. Despite the critical nature of the director of nursing role in long-term care homes, none of the included studies in these reviews focused on specifically examining the structures and processes of the director of nursing role as a unique role that differs from the nursing home administrator and influences work ethos and quality care. The current review will contribute to the growing body of the literature by examining international research in which the director of nursing is exclusively sampled and identifying modifiable structures at the organizational level and processes that may support their leadership role in long-term care homes.

## 2. Aim

The aim of this integrative review is to synthesize the literature related to the director of nursing role through the application of Donabedian's structure-process-outcome quality framework by addressing the research question: What are the structures and processes that support the role of the director of nursing in long-term care homes?

## 3. Methods

### 3.1. Design

An integrative review approach was employed, as it provides the opportunity to examine phenomenon broadly through the inclusion of both empirical and theoretical publications, which are common to nursing research [28, 29]. Cooper's five stages of the integrative review process were applied as follows: problem formulation, literature search, data evaluation, data analysis, and presentation [30].

#### 3.1.1. Theoretical Framework

The theoretical framework underpinning the analysis for this review is Donabedian's structure-process-outcome quality framework [31, 32]. This framework is commonly utilized in research to examine factors influencing quality in healthcare such as program delivery, characteristics of leaders, and burnout amongst care providers [16, 33, 34]. The framework consists of three distinct components—structures, processes, and outcomes. Structures have been described as the physical and organizational setting where care is delivered; processes as the mechanisms and actions allow for healthcare delivery; and outcomes describe the result on individuals and/or communities [31, 32].

For the purposes of this review, we examined structures and processes, defined as characteristics of the individual and organization, and actions of individuals and organizational approaches that impact the director of nursing role, respectively. Outcomes stemming from their performance are outside the scope of this literature review.

### 3.2. Search Strategy

The review assessed electronically available papers that were published between January 2000 and February 2022. The search was limited to this timeframe because long-term care homes changed considerably in 2000, with an increase in resident frailty, integration of technology, and workforce challenges that all impact the director of nursing role [2]. A university health science librarian assisted with the search strategy in five electronic databases: Medline, Embase, CINAHL, AgeLine, and Cochrane Database of Systematic Reviews.  Table S1 in the online appendix describes the full-search strategy.

The inclusion criteria for studies were as follows: (a) focused on the role of the registered nurse in a director of nursing position within the long-term care home with oversight of the nursing department; (b) for qualitative studies or mixed methods studies, described a relationship or theme relating to a structure or process describing or influencing the role; (c) for quantitative studies, reported a relationship between the structure and process and an outcome measure directly related to the role; and (d) published in the English language. The exclusion criteria for studies were as follows: (a) settings that were not long-term care; (b) focused on other executive roles (e.g., administrator); and (c) other reviews, dissertations, conference proceedings, editorials, personal reflections, and commentaries. A snowball search of the reference lists from eligible studies was also completed; from this, 21 studies were identified. However, they were not included in the final review as they did not meet the eligibility criteria.

### 3.3. Data Screening, Extraction, Study Evaluation, and Analysis

#### 3.3.1. Data Screening

Screening of papers took part in two stages using Covidence: at the title/abstract level and at the full-text level. During screening, JF and AW reviewed abstracts and full texts independently. KM and CC resolved any conflicts. After abstract screening, the primary researcher electronically contacted four authors directly to obtain five articles following the Dillman total design survey method [35]. The authors also utilized the university's interlibrary loan services which can access additional closed-access journals beyond the institution's library collection in an attempt to acquire the digital copies of the same five articles and one additional article. From these efforts, three articles were obtained: one from authors and one through interlibrary loan. The remaining four articles were not included as they were not available.

#### 3.3.2. Data Extraction

Data were extracted from each article into a Microsoft Excel table. Study, setting, and sample characteristics are available in Table S3 in the online appendix. To ensure rigor in the data extraction process, JF and AW extracted data independently, and comparison and conflict resolution was performed by JF.

#### 3.3.3. Data Evaluation/Quality Assessment

The quality of each individual study was appraised using the Mixed Methods Assessment Tool (MMAT) [36]. The MMAT tool was selected to be inclusive of all possible research designs. Each study was appraised independently by JF and AW against the corresponding criteria, and KM and CC resolved any conflicts. All articles were retained to support a comprehensive review of the literature [36].

#### 3.3.4. Data Analysis and Presentation

**Figure 1:**
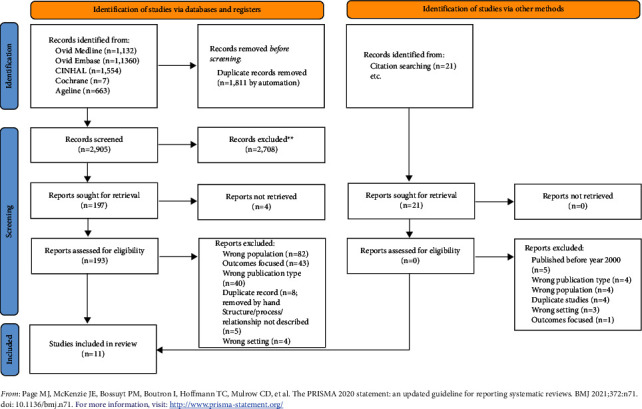
PRISMA illustration.

Data were analyzed by JF by reviewing each article and identifying subthemes that met the descriptions under each structure and process component of the framework. Structures were identified at the individual level to provide insight into characteristics of the director of nursing and at the organizational level to determine factors that were modifiable. The subthemes were then counted to determine the frequency in which they appeared in the literature and were categorized into larger themes as appropriate. The data were then analyzed by comparing commonalities, relationships, and differences to draw conclusions in the review [28, 29]. The results are presented in a PRISMA flow diagram in Figure 1 with tables and thematic narration detailed in Supplementary Materials.

## 4. Results

The initial search yielded 4,716 findings. Following the removal of duplicates, 2,905 papers proceeded to title/abstract screening. After full-text screening, 11 papers were included in this review. Figure 1 includes the PRISMA flow diagram which illustrates the search findings, and Table S2 in the online appendix demonstrates the impact of limiters.

### 4.1. Study Characteristics and Quality Assessment

Five of the 11 studies in this review were quantitative [24, 37–40], and six were qualitative [3, 15, 23, 41–43]. Study and setting characteristics are described in Table S3 in the online appendix.

#### 4.1.1. Sample Characteristics

Sample sizes of the participants ranged from 4 to 247 across the 11 studies. The age of the director of Nursing was reported as a mean in five of the studies, with a mean range of 46–50 years of participant age [3, 15, 23, 37, 38]. The sex of the director of nursing was reported in three of the studies, with females representing 83% to 98.7% of the samples [3, 15, 38]. Educational preparation was reported in six of the studies, with participants holding predominantly a nursing diploma or an associated degree. Current tenure was reported as a mean of 4- 5 years across three studies [23, 24, 37].

Of the 11 studies, directors of nursing were sampled exclusively in three studies [23, 37, 38], and the remaining eight studies included directors of nursing and other personnel including unit managers, nursing home administrators, and staff nurses in their samples.

#### 4.1.2. Quality Assessment

Results of the quality assessment are presented in Tables S4-S5 in the online appendix. The most common limitations were the use of convenience sampling (*n* = 4), small samples (*n* = 2), and low response rates (*n* = 1), which impacted generalizability of findings, and secondary analysis of data (*n* = 3) which limited researchers' ability to confirm interpretation.

### 4.2. Structures Related to the Director of Nursing Role

Five structures were identified in the studies at the individual director of nursing level: years of experience (*n* = 7), level of education (*n* = 5), demonstrated leadership capabilities (*n* = 4), completed certification and/or established linkages with a professional association (*n* = 2), and completed continuing education (*n* = 2). Three structures were identified at the organizational level: physical presence of leadership across the organization (*n* = 4), job description that outlines the director of nursing's key responsibilities and expectations (*n* = 3), and salary (*n* = 1). Tables S6 and S7 in the appendix describe the structures.

#### 4.2.1. Director of Nursing-Level Structures

Previous years of experience was shown to have the greatest effect on the director of nursing role and was the most reported structure across the studies (*n* = 7). This was reported in the form of years of experience as a registered nurse, as a director of nursing, and as a nurse leader/manager. The most commonly reported level of experience was experience as a director of nursing which was reported across six studies [15, 24, 37, 38, 40, 42]. The literature suggests that previous years of experience enhanced the director of nursing's knowledge and ability to lead in complex, unpredictable environments.

Level of education was the second most reported director of nursing structure across five studies [23, 24, 37, 41, 43]. The literature suggests that advanced education beyond a diploma or an associate degree strengthens the director of nursing's ability to strategically plan finite resources and support the interprofessional care team based on advanced knowledge of scopes of practice [23, 43].

Demonstrated leadership capabilities were reported across four studies [3, 15, 39, 41]. Cruttenden [41] observed directors of nursing demonstrate compassion and care through their engagement and support of staff, their belief in ongoing learning, and their desire to be a role model. Siegel et al. [15] noted directors of nursing demonstrating commitment by working long hours to “make a good building” (pg. 220) and advancing their vision through negotiation and influence.

Completed additional licensing certification and/or established linkages with professional associations were reported in two studies [37, 41]. Aroian et al. [37] found directors of nursing that were licensed long-term care administrators scored higher on professional nursing and long-term care leadership than directors of nursing without licensing. In the study by Cruttenden [41], directors of nursing highlighted the importance of gerontological nursing knowledge and being visible in the community and establishing a collaborative insight to help with policy and managerial learning.

Similarly, completed continuing education that furthered professional development was also reported in two studies as helping to further develop competencies and connections [37, 42]. In the study by Aroian et al. [37], continuing education was found to be the most significant structure other than education preparation or experience to support the director of nursing performance.

#### 4.2.2. Organizational-Level Structures

Physical presence of leadership across the organization was an organizational-level structure reported in four studies [15, 23, 38, 42]. This was expressed as the physical presence of leaders in critical roles such as the administrator, department heads, and consulting experts that collaborated with the director of nursing and provided expertise [15, 23, 38, 42].

A job description that outlines the director of nursing key responsibilities and expectations was noted in three studies to support the director of nursing [15, 23, 43]. The literature showed that despite the varied nature of the director of nursing role which, depending on the organization, focused more on either clinical or administrative duties [15, 23, 43], a clear job description made a difference to support the abilities of the director of nursing.

Finally, one study found that a higher salary was an organizational-level structure that impacted the director of nursing. Directors of nursing earning more than $60,000.00 USD were found to be more involved in organization development, professional nursing, and long-term care leadership than directors of nursing who earned less than $60,000.00 USD, who spent more time on resident care and care plans [37].

### 4.3. Processes Related to the Director of Nursing Role

Four processes were identified: nursing home administrator and director of nursing relationship (*n* = 4), availability of onsite continuing education opportunities targeting directors of nursing and support for continuing education (*n* = 4), cultivating relationships and enhancing networks beyond the long-term care home (*n* = 3), and orientation to the role (*n* = 3).  Table S8 available in the online appendix describes process-related themes.

The nursing home administrator and director of nursing relationship was cited across four studies [3, 15, 23, 42]. A relationship built on trust, mutual respect, and collaboration was reported as integral to the director of nursing's ability to perform their role and their sense of autonomy [23]. This was described as the nursing home administrator supporting the director of nursing's continued training and professional development and engaging the director of nursing in decision-making that influences the long-term care home and organizational goals [23].

The availability of onsite continuing education opportunities targeting directors of nursing and support for continued education was a process factor found across four studies [37, 38, 41, 42]. These opportunities were described as onsite workshops, in-services, and meetings on a variety of clinical and nonclinical topics targeted for the director of nursing. Support for continued education was demonstrated through lessening barriers for participation, such as facilitating time away from responsibilities and providing financial reimbursement [37, 38, 41, 42].

Cultivating relationships and enhancing networks beyond the long-term care home setting was a process found across three studies [37, 41, 42]. This was described as a physical presence in the community, participation in meetings, and membership with associations to facilitate peer networks [37, 41]. Peers helped directors of nursing find resources in the community, addressed learning needs related to policies and human resources, and helped manage stress associated with the work environment, role responsibilities, and the regulatory process [37, 41].

Orientation to the director of nursing role was a process identified in three studies [23, 42, 43]. Furthermore, the experiences of directors of nursing that received support from parting directors of nursing, nursing home administrators, or associate directors of nursing were more successful in their transition and orientation to their role and in their overall performance than those without this support [23].

## 5. Discussion

To our knowledge, this is the first review to focus exclusively on the director of nursing, rather than as part of a subset of the sampled population with results describing the collective experiences of medical directors, nursing home administrators, and nonnursing leaders in long-term care. The literature examining the role of the director of nursing in long-term care describes a disconnect between the minimal qualifications required to perform the role and actual duties, responsibilities, and reach impacting their duration in the role. This review describes several modifiable structures at the organizational level and processes that may support the director of nursing in their role which can impact the high turnover of directors of nursing who are often in their roles for less than a year and those with higher levels of education leaving the role sooner [20, 21, 24, 37, 43]. For those who remain in the position longer, the literature shows that the first five years of the director of nursing role is critical. The identified structures and processes can be targeted within this timeframe to enable directors of nursing ability to effectively lead, positively influence job satisfaction, and remain in the role over time. For example, effective staff orientation during the director of nursing transitions has shown positive effects on their experiences in the role [23]. Skills and knowledge-based competencies including expertise in gerontology that increase directors of nursing confidence and enhance the effectiveness of the director of nursing leadership across long-term care homes can only develop over time.

The organizational-level structures that influence the director of nursing role were the physical presence of leaders across the organization in roles such as nursing home administrators and department directors, a clear job description that outlines roles and responsibilities, and salary. Research shows that the continual pull on the director of nursing role to take on roles and responsibilities outside of nursing may result in job dissatisfaction and further perpetuate laissez-faire or a transactional approach to leadership, with a focus on tasks and not on relationships [23, 24, 39, 43]. Hence, the presence and support of these nursing home administrators and other leaders serve as valuable support to help the director of nursing advance transformative change across the long-term care home. Additional research on the role of the director of nursing within quality assurance and the interprofessional context, including but not limited to the role of the medical director and nurse practitioner, needs to be conducted.

Furthermore, a clear job description assists in determining education, training, certification, and competencies required for the role. It is clear from the literature that expectations of the director of nursing role vary between long-term care homes with some directors of nursing performing their roles focusing on clinical care and others focusing on staffing and responding to regulatory requirements which require leadership and managerial competencies [15, 43]. Future research is needed to determine which type of role leads to better staff and resident outcomes.

The nursing home administrator plays an integral role in the mentorship, coaching, and support of the director of nursing and the directors of nursing effectiveness in leading within the long-term care home. A strong relationship rooted in trust and respect is foundational to the director of nursing authority and decision-making ability. Fleming and Kayser-Jones [23] also recognize the power dynamics of the relationship, as the director of nursing is hired by and reports directly to the nursing home administrator, suggesting that not all directors of nursing may be comfortable with asking the nursing home administrator for support. Understanding the nuances of the nursing home administrator/director of nursing relationship and the impact this has on the director of nursing retention is another area for future research.

### 5.1. Strengths and Limitations

The review has several strengths. First, the search strategy included several databases and snowball searching of relevant articles. Second, the review was guided by expert researchers with advanced knowledge in the theoretical model, the integrative review process, and the long-term care home sector. A team approach was used throughout the integrative review process to enhance the quality of the review [44]. One limitation of the review is that our definition of the director of nursing required an RN to fulfill the position which is common practice in North America; however, this may have excluded articles focused on the director of nursing role held by another profession. The quality of the results is limited by the quality of the studies included, which includes a relatively small number of studies that were found to be relevant. Furthermore, not all authors reported findings equally for each of the sampled populations (e.g., nursing home administrator education was reported, but director of nursing education was not reported) [40]. Our search was also limited to electronically available peer-reviewed articles and did not include grey literature such as government reports or dissertations; as such, we may have missed examples of other supports that enhance the director of nursing performance in their role. Many of the included studies in this review had methodology weaknesses in their design, thus limiting generalizability of findings. Lastly, our review findings and implications are limited to the North American context.

## 6. Conclusions

The 11 articles synthesized in this integrative review present the structures and processes that support the director of nursing role in long-term care homes. The findings of this review have implications for leaders, decision-makers, practice experts, and researchers and identify specific structures and processes to focus interventions and innovations to support the director of nursing. Inadequate preparation, poor hiring practices, lack of orientation, unclear expectations, and increases in regulatory practices and stress as described across the studies can impact recruitment and retention of competent directors of nursing to the role, further perpetuating the human health resource crisis.

### 6.1. Implications for Nursing Management

There have been long-standing issues with the nursing leadership workforce in long-term care that have only worsened during the pandemic; this coupled with an increasing need for long-term care, concerns around quality, and a health human resource crisis made for a sector that is expected to deliver care to vulnerable populations while experiencing tremendous pressure. The director of nursing role within long-term care homes is critical to the overall operations impacting workforce stability and the delivery of quality care. Given the impact of this nursing leadership role, investment in the director of nursing role in the form of orientation, mentoring, and support for continued professional development is also an investment in the staff and the residents that call long-term care their home.

## Data Availability

The data used to support the findings of this study are available from the corresponding author upon request.

## Supplementary Materials

Supplementary Materials Table S1: search terms and search strategy for the electronic database. Table S2: overview of imported articles. Table S3: description of the included studies by study, setting, and sample characteristics (*N* = 11). Table S4: quality assessment of qualitative studies using MMAT (*N* = 6). Table S5: quality assessment of quantitative studies using MMAT (*N* = 5). Table S6: structure-related themes at the individual level. Table S7: structure-related themes at the organizational level. Table S8: process-related themes.
