# Supplementary material for: Examining the Director of Nursing Role in Long-Term Care: An Integrative Review
Source: J Nurs Manag. 2023 Nov 6;2023:8200746. doi: 10.1155/2023/8200746 (PMC11918960; doi:10.1155/2023/8200746)
Supplement: Supplementary Materials — Table S1: search terms and search strategy for the electronic database. Table S2: overview of imported articles. Table S3: description of the included studies by study, setting, and sample characteristics (N = 11). Table S4: quality assessment of qualitative studies using MMAT (N = 6). Table S5: quality assessment of quantitative studies using MMAT (N = 5). Table S6: structure-related themes at the individual level. Table S7: structure-related themes at the organizational level. Table S8: process-related themes. [file 8200746.f1.doc]

**Supplementary Material Description**

Table S1 – Search terms and search strategy for electronic database

Table S2 – Overview of imported articles

Table S3 – Description of included studies by study, setting, and sample characteristics (*N = 11)*

Table S4 – Quality assessment of qualitative studies using the MMAT (*N = 6)*

Table S5 – Quality assessment of quantitative studies using the MMAT (*N = 5)*

Table S6 – Structure related themes at the individual level

Table S7 – Structure related themes at the organizational level

Table S8 – Process related themes

**Supplementary Materials**

**Supplementary Table S1 - *Search Terms and Search Strategy for Electronic Databases***

| **Ovid Medline Database** | |
| --- | --- |
| **Setting** | **MESH**  Long Term Care/  Homes for the Aged/  Residential Facilities/  Nursing Homes/ or Intermediate Care Facilities  **Title/Abstract/Key Word**  ((extended care or assisted living or residential or intermediate care) adj2 (facilit*)).tw.kf  ((nursing or residential care or residential aged care) adj2 home*)).tw,kf.  ((assisted living or residential aged) adj2 (care)).tw,kf  (long term care or longterm care).tw,kf  home* for the aged.tw,kf |
| **Role** | **MESH**  Nurse Administrators/  Health Facility Administrators/  **Title/Abstract/Key Word**  ((director* or administrator*) adj2 (nurs* or care or health facilit* or home* or nursing home*)).tw,kf  ((nursing or nursing home) adj2 (manage*)).tw,kf |
| **Search** | 1 Long-Term Care/  2 Homes for the Aged/  3 Assisted Living Facilities/  4 Residential Facilities/  5 nursing homes/ or intermediate care facilities/  6 ((extended care or assisted living or residential or intermediate care) adj2 facilit*).tw,kf.  7 ((nursing or residential care or residential aged care) adj2 home*).tw,kf.  8 ((assisted living or residential aged) adj2 care).tw,kf.  9 (long term care or longterm care).tw,kf.  10 home* for the aged.tw,kf.  11 1 or 2 or 3 or 4 or 5 or 6 or 7 or 8 or 9 or 10  12 Nurse Administrators/  13 Health Facility Administrators/  14 ((director* or administrator*) adj2 (nurs* or care or health facilit* or home* or nursing home*)).tw,kf.  15 ((nursing or nursing home) adj2 manage*).tw,kf.  16 12 or 13 or 14 or 15  17 11 and 16  18 limit 17 to yr="2000 -Current"  19 limit 18 to english language |
| **Ovid Embase Database** | |
| **Setting** | **MESH**  Long Term Care/  Homes for the Aged/  Assisted Living Facility/  Nursing Home/  Exp Residential Home/  **Title/Abstract/Keyword**  ((extended care or assisted living or residential or ((intermediate care) adj2 (facilit*)).ti,ab,kw.  ((nursing or for the aged or resident care or residential aged care) adj2 (home*)).ti,ab,kw.  ((assisted living or residential aged) adj2 (care)).ti,ab,kw.  (long term care or longterm care).ti,ab,kw. |
| **Role** | **MESH**  Nurse Administrator/  **Title/Abstract/Keyword** ((director* or administrator*) adj2 (nur* or care or facilit* or nursing home*)).ti,ab,kw.  ((nursing or nursing home) adj2 (manage*)).ti,ab,kw. |
| **Search** | 1 long term care/  2 home for the aged/  3 assisted living facility/  4 nursing home/  5 exp residential home/  6 ((extended care or assisted living or residential or intermediate care) adj2 facilit*).ti,ab,kw.  7 ((nursing or for the aged or resident care or residential aged care) adj2 home*).ti,ab,kw.  8 ((assisted living or residential aged) adj2 care).ti,ab,kw.  9 (long term care or longterm care).ti,ab,kw.  10 1 or 2 or 3 or 4 or 5 or 6 or 7 or 8 or 9  11 nurse administrator/  12 ((director* or administrator*) adj2 (nur* or care or facilit* or nursing home*)).ti,ab,kw.  13 ((nursing or nursing home) adj2 manage*).ti,ab,kw.  14 11 or 12 or 13  15 10 and 14  16 limit 15 to yr="2000 -Current"  17 limit 16 to english language |
| **CINHAL Plus with Full Text Database** | |
| **Setting** | **MESH**  (MH "Residential Facilities") OR (MH "Nursing Homes") OR (MH "Assisted Living") OR (MH "Long Term Care")  **Title/Abstract**  TI ( ((extended care or assisted living or residential or intermediate care) N2 (facilit*)) ) OR AB ( ((extended care or assisted living or residential or intermediate care) N2 (facilit*)) )  TI ( ((nursing or resident care or residential aged care) N2 (home*)) ) OR AB ( ((nursing or resident care or residential aged care) N2 (home*)) )  TI ( ((assisted living or residential aged) N2 (care) ) OR AB ( ((assisted living or residential aged) N2 (care) )  TI ( (long term care or longterm care) ) OR AB ( (long term care or longterm care) )  TI "home* for the aged" or AB "home* for the aged" |
| **Role** | **MESH**  (MH "Health Facility Administrators") OR (MH "Nurse Administrators") OR (MH "Nursing Management")  **Title/Abstract** TI ( ((director* or administrator*) N2 (nurs* or care or health facilit* or nursing home*)) ) OR AB ( ((director* or administrator*) N2 (nurs* or care or health facilit* or nursing home*))  TI ( ((nursing or nursing home*) N2 (manage*) ) ) OR AB ( ((nursing or nursing home*) N2 (manage*) ) ) |
| **Search** | | S14 | S10 AND S11 (limiter year) | | --- | --- | | S13 | S10 AND S11 (limiter English) | | S12 | S10 AND S11 | | S11 | S7 OR S8 OR S9 | | S10 | S1 OR S2 OR S3 OR S4 OR S5 OR S6 | | S9 | TI ( ((nursing or nursing home*) N2 (manage*) ) ) OR AB ( ((nursing or nursing home*) N2 (manage*) ) ) | | S8 | TI ( ((director* or administrator*) N2 (nurs* or care or health facilit* or nursing home*)) ) OR AB ( ((director* or administrator*) N2 (nurs* or care or health facilit* or nursing home*)) ) | | S7 | (MH "Health Facility Administrators") OR (MH "Nurse Administrators") OR (MH "Nursing Management") | | S6 | TI "home* for the aged" or AB "home* for the aged" | | S5 | TI ( (long term care or longterm care) ) OR AB ( (long term care or longterm care) ) | | S4 | TI ( ((assisted living or residential aged) N2 (care) ) OR AB ( ((assisted living or residential aged) N2 (care) ) | | S3 | TI ( ((nursing or resident care or residential aged care) N2 (home*)) ) OR AB ( ((nursing or resident care or residential aged care) N2 (home*)) ) | | S2 | TI ( ((extended care or assisted living or residential or intermediate care) N2 (facilit*)) ) OR AB ( ((extended care or assisted living or residential or intermediate care) N2 (facilit*)) ) | | S1 | (MH "Residential Facilities") OR (MH "Nursing Homes") OR (MH "Assisted Living") OR (MH "Long Term Care") | |
| **Cochrane Database** | |
| **Setting** | **MESH**  [mh ^“long-term care”]  [mh ^“homes for the aged”]  [mh ^“assisted living facilities”]  [mh ^“residential facilities”]  [mh ^“intermediate care facilities”]  **Title/Abstract/Keyword**  ((extended care or assisted living or resident or intermediate care) NEAR/2 (facility*)):ti,ab,kw  ((nursing or residential or residential aged care) NEAR/2 (home*)):ti,ab,kw  (long term care or longtermcare):ti,ab,kw  (home* for the aged):ti,ab,kw |
| **Role** | **MESH**  [mh ^“nurse administrators”]  [mh ^“health facility administrators”]  **Tilte/Abstract/Keyword**  ((director* or administrator*) NEAR/2 (nurs* or care or health facility* or home* or nursing home*)):ti,ab,kw  ((nursing or nursing home) NEAR/2 (manage*)):ti,ab,kw |
| **Ageline Database** | |
| **Setting** | **MESH**  DE "Long Term Care"  DE "Homes for the Elderly"  DE "Assisted Living Facilities"  DE "Nursing Homes"  **Title/Abstract**  TI ( "extended care facilit*" OR "assisted living facilit*" or "residential facilit*" or "intermediate care facilit*" ) OR AB ( "extended care facilit*" OR "assisted living facilit*" or "residential facilit*" or "intermediate care facilit*" ) OR ( ID "extended care facilit*" OR "assisted living facilit*" or "residential facilit*" or "intermediate care facilit*" )  TI ( "nursing home*" OR "residential care home*" OR "residential aged care home*" ) OR AB ( "nursing home*" OR "residential care home*" OR "residential aged care home*" ) OR ( ID "nursing home*" OR "residential care home*" OR "residential aged care home*" )  TI ( "assisted living care" OR "residential aged care" OR "long term care" OR "longterm care" OR "home* for the aged" ) OR AB ( "assisted living care" OR "residential aged care" OR "long term care" OR "longterm care" OR "home* for the aged" ) OR ( ID "assisted living care" OR "residential aged care" OR "long term care" OR "longterm care" OR "home* for the aged" ) |
| **Role** | **MESH**  DE "Long Term Care Administration"  DE "Nursing Home Administrators"  **Title/Abstract**  TI ( "director* of care" OR "director* of nursing" ) OR AB ( "director* of care" OR "director* of nursing" ) OR ( ID "director* of care" OR "director* of nursing" )  TI ( "health facilit* director*" OR "home director*" OR "nursing home* director*" ) OR AB ( "health facilit* director*" OR "home director*" OR "nursing home* director*" ) OR ( ID "health facilit* director*" OR "home director*" OR "nursing home* director*" )  TI ( "nurse administrator* OR "health facilit* administrator*" OR "home administrator*" OR "nursing home administrator*" ) OR AB ( "nurse administrator* OR "health facilit* administrator*" OR "home administrator*" OR "nursing home administrator*" ) OR ( ID "nurse administrator* OR "health facilit* administrator*" OR "home administrator*" OR "nursing home administrator*" )  TI ( "nursing home manager*" OR "nurse manager*" ) OR AB ( "nursing home manager*" OR "nurse manager*" ) OR ( ID "nursing home manager*" OR "nurse manager*" ) |
| **Search** | **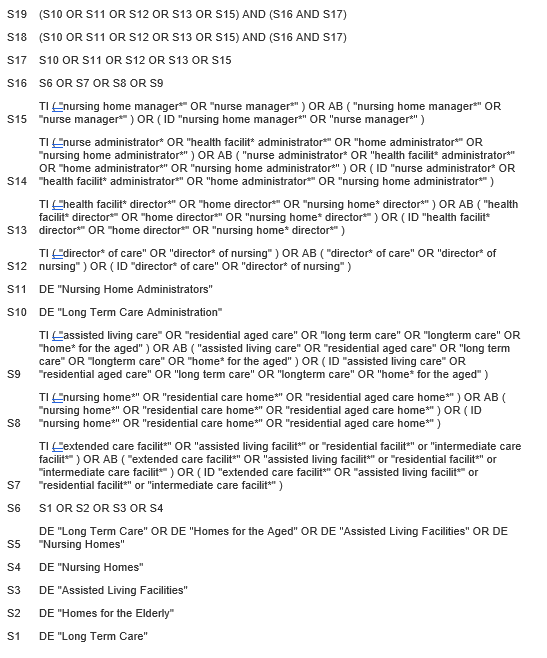** |

***Supplementary Table S2 - Overview of Imported Articles***

| **Database** | **Results** | **Limiter #1**  **(Year 2000)** | **Limiter #2**  **(English language)** | **Uploaded to Covidence** |
| --- | --- | --- | --- | --- |
| **Ovid Medline** | 1993 | 1193 | 1132 | 1132 |
| **Ovid Embase** | 2091 | 1420 | 1360 | 1360 |
| **CINHAL** | 2022 | 1615 | 1554 | 1554 |
| **Cochrane** | 7 | 7 | 7 | 7 |
| **Ageline** | 1458 | 663 | 663 | 663 |
| **TOTAL:** |  |  |  | 4716 |

**Supplementary Table S3 - *Study, Setting, and Sample Characteristics***

|  |  |  |  | **Setting Characteristics** | | **Sample Characteristics** | | | | | | | |
| --- | --- | --- | --- | --- | --- | --- | --- | --- | --- | --- | --- | --- | --- |
| Author; Year | Country | Aim | Design | Facility Size | Facility Ownership | Sample Size | Mean Age | Sex | Highest Level of Education | Years as an RN[[1]](#footnote-2) | Years in Current Role | Years with Leadership/ Management Experience | DON[[2]](#footnote-3) included as part of larger study |
| Aroian;  2000 | USA  New England | To replicate and geographically extend a descriptive survey conducted in North Carolina by Heine (1995) to determine the roles and responsibilities of DONs in LTCNFs as well as their educational preparation, current professional credentials, and educational needs within the New England States | Quantitative  Descriptive Cross-Sectional Survey | N/A[[3]](#footnote-4) | N/A | 247 | M=46.92 (SD=8.14); range 26 to 68 | N/A | N=247 N=71 (28.7%) diploma (nurse)  N=45 (18.2%) associate degree (nurse) N=1 (0.4%) associate degree (other)  N=49 (19.8%) bachelor (nurse) N=28 (11.3%) bachelor (other)  N=20 (8.1%) masters (nurse) N=32 (13%) masters (other) N=1 (0.4%) not reported | M=23.17 (SD 9.30); 1 to 44 years | M=5.05 (SD=4.83); range 1 month to 26 years | M=11.96 (SD=6.65); 1 to 30 years | No |
| Cruttenden; 2006 | Canada  New Brunswick | To understand the strengths and learning needs of staff in order to plan appropriate learning opportunities for nurses providing care in nursing homes. | Qualitative – Ethnography | range 38-196 | N/A | 4 | N/A | N/A | N/A | N/A | 2 new;  2 longer standing | N/A | Yes |
| Daly;  2015 | U.S.A.  Iowa | To conduct a needs assessment (knowledge deficit) of DONs in IOWA nursing homes in relation to caring for patients with Behavioural and Psychological symptoms of dementia behaviours and how to best receive this additional training. | Quantitative - Descriptive Cross-Sectional Survey | N/A | N/A | 160 | N= 153 M=45.4 (SD=9.7); range 23 to 66 | N=156 N=154 (98.7%) female; N=2 (1.3%) male | N=156 N=97 (62.2%) associate degree N=41 (26.3%) bachelor N=14 (9.0%) diploma N=6 (3.9%) MS N=1 (0.6%) PhD | N=156 M=20.4 (SD 9.9); 2 to 44 years | N/A | N/A | No |
| Fleming; 2008 | U.S.A.  West Coast – 5 States | To describe and analyze the phenomenon of leadership from the perspective of DONs.  How DONs assume leadership and what factors influence their leadership. | Qualitative - Ethnography | N/A | Private/ For Profit Corporate multi-facility chains | 10 | M=45.6 (SD=9.2); range 33 to 62 | N/A | N=10  N=1 (10%) diploma N=1 (10%) associate degree N=6 (60%) bachelor  N=2 (20%) masters Public Health | M=20.8 (SD 8.12); 8 to 40 years | M=4.90 (SD 4.35); 1 to 14 years | N/A | No |
| Forbes-Thompson; 2006 | U.S.A.  Kansas | To understand the interplay between NH organizational processes and characteristics of the DON and NHA | Quantitative – Descriptive Cross-Sectional Survey | M = 80 | N=222 NFP = 44% FP = 56% urban = 32% rural = 68% | N/A | N/A | N/A | Diploma or associate degree =64% | N/A | M=4.26; (SD 4.49) M=2.15; (SD 3.22) years of experience priori to current position | N/A | Yes |
| Poels;  2020 | Belgium  6 NH Across Belgium | To explore leadership styles in nursing homes based on well-described concepts and outcomes of the present leadership styles. | Quantitative Descriptive Cross-Sectional Survey | N/A | N/A | N/A | N/A | N/A | N/A | N/A | N/A | N/A | Yes |
| Resnick; 2009 | U.S.A. | To understand the key characteristics of the leadership team, and to examine if differences in these factors exist between FP and NFP NHs. | Quantitative Descriptive Cross-Sectional Survey | 3-49 = 13.9% 50-99 = 37.3% 100 -199 - 42.5% >200 = 6.2% | N=1174 NFP (38.5%) and FP (61.5%) 54.2% part of a chain; 45.8% not part | N/A | N/A | N/A | N/A | N/A | Tenure at Current Facility <1 year = 30.4% 1-4 years = 46.8% 5-9 years = 13.8% >10 years = 9.1% | <1 year = 11.3%  1-4 years = 38.3%  5-9 years = 24.3%  >10 years = 23.8% | Yes |
| Siegel; 2012 | U.S.A.  15 States | To describe and examine the roles and responsibilities of DONs as perceived by current/previous DONs and Nursing Home Administrators | Qualitive Nonspecific, Primary Analysis | N/A | N/A | 11 DON; 22/29 DON (incl. past) | N= 24 M=49.9; range 34 to 64 | N=24 (83%) female N=5 (17%) male | N=11  N=2 (18%) associate degree N=2 (18%) ass. degree + some college N=3 (27%) bachelor N=4 (36%) masters | N/A | N/A | N/A | Yes |
| Siegel & Sikma; 2015 | U.S.A.  15 States | To describe RNs transitions into their first nursing home DON positions, including hiring practices and role development. | Qualitative -Nonspecific, Secondary Analysis | N/A | N/A | Info from parent study | Info  from parent study | Info from parent study | Info  from parent study | N/A | N/A | N/A | Yes |
| Siegel; 2015 | U.S.A.  15 States | To describe the nursing service demands-resources tensions that DONs face on a day-to-day basis and the tactics they use to secure and manage resources for the nursing department. | Qualitative - Nonspecific, Secondary Analysis | N/A | N/A | Info from parent study | N= 29 M=49.9; range 34 to 64 | N=29 (83%) female N=5 (17%) male | N/A | N/A | N/A | N/A | Yes |
| Siegel; 2018 | U.S.A.  15 States | To describe the challenges faced by nursing home DONs leading and managing a team nursing approach, including consideration of scope of practice, delegation, and supervision regulations, and related policy implications. | Qualitative - Nonspecific, Secondary Analysis | N/A | N/A | Info from parent study | Info  from parent study | Info from parent study | N=11  N=2 (18%) associate degree N=2 (18%) ass. degree + some college N=3 (27%) bachelor N=4 (36%) masters | N/A | N/A | N/A | Yes |

**Supplementary Table S4 - *Mixed Methods Appraisal Results for Qualitative Studies***

|  | |  |  |  |  |  |  |  |
| --- | --- | --- | --- | --- | --- | --- | --- | --- |
| **Study Info:** | | **Screening Questions:** | | **Qualitative Methodological Criteria:** | | | | |
| First author & Year | Type of Study | S1 - Are there clear research questions? | S2 - Do the collected data allow to address the research questions? | 1.1 Is the qualitative approach appropriate to answer the research question? | 1.2 Are the qualitative data collection methods adequate to address the research question? | 1.3 Are the findings adequately derived from the data? | 1.4 Is the interpretation of results sufficiently substantiated by data? | 1.5 Is there a coherence between qualitative data sources, collection, analysis and interpretation |
| Cruttenden, 2006 | Qualitative - Ethnography, p | yes | yes | yes | yes | yes | yes | yes |
| Fleming et al., 2008 | Qualitative - Ethnography, p | yes | yes | yes | yes | yes | yes | yes |
| Siegel et al., 2018 | Qualitative - Descriptive, s | yes | yes | yes | yes | yes | yes | yes |
| Siegel & Sikma, 2015 | Qualitative - Descriptive, s | yes | yes | yes | yes | yes | yes | yes |
| Siegel et al., 2012 | Qualitative - Phenomenology, p | yes | yes | yes | yes | yes | yes | yes |
| Siegel et al., 2015 | Qualitative - Phenomenology, s | yes | yes | yes | yes | yes | yes | yes |

**Legend**

P – primary

S – secondary

**Supplementary Table S5 - *Mixed Method Appraisal Results for Quantitative Studies***

|  | |  |  |  |  |  |  |  |
| --- | --- | --- | --- | --- | --- | --- | --- | --- |
| **Study Info:** | | **Screening Questions:** | | **Quantitative Descriptive Methodological Criteria:** | | | | |
| First author & Year | Type of Study | S1 - Are there clear research questions? | S2 - Do the collected data allow to address the research questions? | 4.1 Is the sampling strategy relevant to address the research question? | 4.2 Is the sample representative of the target population? | 4.3 Are the measurements appropriate? | 4.4 Is the risk of nonresponse bias low? | 4.5 Is the statistical analysis appropriate to answer the research question? |
| Aroian et al., 2000 | Quantitative - survey, p | yes | yes | yes | yes | yes | can't tell (26% response rate) | yes |
| Daly et al., 2015 | Quantitative - survey, p | yes | yes | yes | yes | yes | can't tell (37% response rate) | yes |
| Forbes-Thompson et al., 2006 | Quantitative - survey, p | yes | yes | yes | yes | yes | yes | yes |
| Poels et al., 2020 | Quantitative - survey, p | yes | yes | yes | yes | yes | yes | yes |
| Resnick et al., 2009 | Quantitative - survey, p | yes | yes | yes | yes | yes | yes | yes |

**Legend:**

P – primary

S – secondary

***Supplementary Table S6*** *- Structure Related Themes at the Individual Level*

| **Major Theme** | Higher Level of Education | Years of  Experience | | | Completed Certification and/or Established Linkages with a Professional Association | Completed Continuing Education | Demonstrated Leadership Capabilities | |
| --- | --- | --- | --- | --- | --- | --- | --- | --- |
| **Sub-Themes** | Level of Education | Years as an RN | Years as a DON | Experiencing in Leadership/ Management | Completed Certification and/or Established Linkages with a Professional Association | Completed Continuing Education | Vision, Caring Attitude, Confidence | Role Responsibility/ Commitment |
| Aroian et al., 2000 | X |  | X | X | X | X |  |  |
| Cruttenden, 2006 | X |  |  |  | X |  | X |  |
| Daly et al., 2015 |  | X | X |  |  |  |  |  |
| Fleming et al., 2008 | X |  |  | X |  |  |  |  |
| Forbes-Thompson et al., 2006 | X |  | X | X |  |  |  |  |
| Poels et al., 2020 |  |  |  |  |  |  |  | X |
| Resnick et al., 2019 |  |  | X |  |  |  |  |  |
| Siegel et al., 2018 | X |  |  |  |  |  |  |  |
| Siegel & Sikma, 2015 |  |  | X |  |  | X |  |  |
| Siegel et al., 2012 |  |  | X |  |  |  | X |  |
| Siegel et al., 2015 |  |  |  |  |  |  | X |  |
| **TOTALS FOR SUBTHEMES:** | 5 | 1 | 6 | 3 | 2 | 2 | 3 | 1 |
| **TOTAL STUDIES:** | **5 studies** | **7 studies** | | | **2 studies** | **2 studies** | **4 studies** | |

***Supplementary Table S7*** *- Structure Related Themes at the Organizational Level*

| **ORGANIZATIONAL LEVEL - STRUCTURES** | | | | |
| --- | --- | --- | --- | --- |
| **Major Theme** | Clear Job Description | Presence of Leadership Across the Organization | | Salary |
| **Sub-Themes** | Job Description/ Clear Role | Department Level | Availability of Consulting Professionals  (e.g., Pharmacy/ Psychiatrist, HR[[4]](#footnote-5)¶) | Salary |
| Aroian et al., 2000 |  |  |  | X |
| Cruttenden, 2006 |  |  |  |  |
| Daly et al., 2015 |  |  | X |  |
| Fleming et al., 2008 | X | X |  |  |
| Forbes-Thompson et al., 2006 |  |  |  |  |
| Poels et al., 2020 |  |  |  |  |
| Resnick et al., 2019 |  |  |  |  |
| Siegel et al., 2018 | X |  |  |  |
| Siegel & Sikma, 2015 |  | X | X |  |
| Siegel et al., 2012 | X | X |  |  |
| Siegel et al., 2015 |  |  |  |  |
| **TOTALS FOR SUBTHEMES:** | 3 | 3 | 2 | 1 |
| **TOTAL STUDIES:** | **3 studies** | **4 studies** | | **1 study** |

**Supplementary Table S8 - *Process Related Themes***

|  |  | **PROCESS THEMES** | | |  | |  |
| --- | --- | --- | --- | --- | --- | --- | --- |
| **Major Theme** | NHA and DON Role and Relationship | Availability of Onsite Targeted DON CE[[5]](#footnote-6)†† and  Organizational Support for CE | | | Cultivating Relationships and Enhancing Networks Beyond the LTC Home[[6]](#footnote-7)‡‡ | | Orientation to the Role |
| **Sub-Themes** | Relationship with NHA (incl. coaching, mentoring, supporting) | Onsite CE | Financial Resources | Time Related Resources | Hiring Practices/ Orientation | Attendance/Presence Meetings/Professional Associations | Hiring Practices/  Orientation |
| Aroian et al., 2000 |  | X | X |  |  |  |  |
| Cruttenden, 2006 |  | X |  |  |  | X |  |
| Daly et al., 2015 |  | X |  |  |  |  |  |
| Fleming et al., 2008 | X |  |  |  | X |  | X |
| Forbes-Thompson et al., 2006 |  |  |  |  |  |  |  |
| Poels et al., 2020 |  |  |  |  |  |  |  |
| Resnick et al., 2019 |  |  |  |  |  |  |  |
| Siegel et al., 2018 |  |  |  |  | X |  | X |
| Siegel & Sikma, 2015 | X |  | X | X | X | X | X |
| Siegel et al., 2012 | X |  |  |  |  |  |  |
| Siegel et al., 2015 | X |  |  |  |  |  |  |
| **TOTALS FOR SUBTHEMES:** | 4 | 3 | 2 | 1 | 3 | 2 | 3 |
| **TOTAL STUDIES:** | **4 studies** | **4 studies** | | | **3 studies** | | **3 studies** |

1. Registered Nurse [↑](#footnote-ref-2)
2. Director of Nursing [↑](#footnote-ref-3)
3. Not Available [↑](#footnote-ref-4)
4. ^¶^ Human Resources [↑](#footnote-ref-5)
5. ^††^ Continuing education [↑](#footnote-ref-6)
6. ^‡‡^ Long-term care home(s) [↑](#footnote-ref-7)
